# Effects of supplemental vitamin D and calcium on markers of proliferation, differentiation, and apoptosis in the normal colorectal mucosa of colorectal adenoma patients

**DOI:** 10.1371/journal.pone.0208762

**Published:** 2018-12-17

**Authors:** Yasheen Gao, Caroline Y. Um, Veronika Fedirko, Robin E. Rutherford, March E. Seabrook, Elizabeth L. Barry, John A. Baron, Roberd M. Bostick

**Affiliations:** 1 Department of Epidemiology, Emory University, Atlanta, Georgia, United States of America; 2 Winship Cancer Institute, Emory University, Atlanta, Georgia, United States of America; 3 Division of Digestive Diseases, Department of Medicine, School of Medicine, Emory University, Georgia, United States of America; 4 Consultants in Gastroenterology, West Columbia, South Carolina, United States of America; 5 Department of Epidemiology, Geisel School of Medicine at Dartmouth, Lebanon, New Hampshire, United States of America; 6 Department of Medicine, Geisel School of Medicine at Dartmouth, Lebanon, New Hampshire, United States of America; 7 University of North Carolina School of Medicine, Chapel Hill, North Carolina, United States of America; Columbia University, UNITED STATES

## Abstract

To clarify the roles of vitamin D and calcium as potential chemopreventive agents against colorectal cancer in humans, and to develop “treatable”, pre-neoplastic, phenotypic biomarkers of risk for colorectal neoplasms, we estimated the effects of supplemental vitamin D_3_ (1,000 IU/day [25 μg/day]) and calcium (1,200 mg/day), alone and in combination, on biomarkers of proliferation (mib-1), differentiation (p21), and apoptosis (bax [apoptosis-promoting] and bcl-2 [apoptosis-inhibiting]), in the normal-appearing rectal mucosa in a subsample of participants (n = 104) in a larger randomized, double-blind, placebo-controlled clinical trial among colorectal adenoma patients. The biomarkers were measured in rectal biopsies at baseline and after one year of follow up, using automated immunohistochemistry and quantitative image analysis. In the vitamin D plus calcium group relative to control, in the crypt differentiation zone (upper 40% of crypts), mib-1 expression decreased 24% (P = 0.28); p21 expression alone and relative to mib-1 expression increased 29% (P = 0.06) and 73% (P = 0.06), respectively; and bax expression relative to mib-1 expression increased 58% (P = 0.21). The estimated vitamin D alone treatment effects were similar but of lesser magnitudes, and those for calcium alone were mixed. All estimated treatment effects on bcl-2 expression were close to the null. These pilot study results support further investigation of whether 1) vitamin D and calcium promote colorectal epithelial cell differentiation, reduce proliferation, and promote apoptosis in the normal-appearing human colorectal mucosa, 2) vitamin D and calcium act as chemopreventive agents against colorectal neoplasms, and 3) mib-1, p21, and bax are potential “treatable”, pre-neoplastic, biomarkers of risk for colorectal neoplasms.

## Introduction

Colorectal cancer remains the second leading cause of cancer death in the United States, and mortality has only modestly declined in recent years despite advances in screening, treatment, and prevention [1]. Furthermore, recent studies found an increasing burden of colorectal cancer among younger adults [2]. Therefore, the development of treatable pre-neoplastic biomarkers of risk for colorectal neoplasms is needed for assessing and managing colorectal cancer risk and for developing chemopreventive agents against the disease.

The efficacy of calcium and vitamin D as chemopreventive agents against colorectal cancer is supported by strong biological plausibility and animal experimental and human evidence [3–5]. Proposed mechanisms of calcium against colorectal cancer include binding to bile acids and fatty acids to protect colonocytes from their toxic and mutagenic effects [6]; direct regulation of the cell cycle via binding with the calcium-sensing receptor, resulting in suppression of proliferation and promotion of differentiation and apoptosis [4, 7, 8]; inhibition of oxidative DNA damage [4]; and modification of colorectal cancer-related cell signaling pathways [4]. Proposed mechanisms of vitamin D against colorectal cancer include modulation of more than 200 genes involved in, among others, bile acid metabolism, cell cycle regulation, cell adhesion, and growth factor signaling [7].

Calcium intakes and circulating 25-OH-vitamin D (25[OH]D) concentrations have been consistently associated with lower risk for colorectal neoplasms [3–5], and calcium supplementation reduced colorectal adenoma recurrence in two large, randomized, controlled trials [9, 10]. In a previous pilot, randomized, controlled trial in sporadic colorectal adenoma patients (n = 92), we found that supplemental calcium (2,000 mg/day) and vitamin D_3_ (800 IU/day [20 μg/day]) over six months increased the expression of p21 (biomarker of differentiation) [11] and bax (biomarker of apoptosis promotion) [12] in the normal-appearing colorectal mucosa, and in a full-scale trial (n = 193) calcium supplementation statistically significantly shifted downward the colorectal crypt proliferation zone [13]. Inconsistent with these various findings, in a third, large, randomized, controlled trial, there was no overall effect of calcium 1,200 mg/day and/or vitamin D_3_ 1,000 IU/day (25 μg/day) over 3–5 years on colorectal adenoma recurrence [14]. However, in a subset (n = 104) of participants in that trial, we estimated that vitamin D_3_, alone or together with calcium, modified APC, β-catenin, and E-cadherin (all three involved in the APC colon carcinogenesis pathway) expression in directions hypothesized to reduce risk for colorectal neoplasms [15]. All of these findings taken together suggest that higher intakes of calcium and higher vitamin D exposure may reduce risk for colorectal neoplasms, that they may do so primarily via mechanisms most relevant in the early stages of the long process of neoplastic development, and that colorectal adenoma recurrence trials with only 3–5 years of treatment and follow up may be of insufficient duration.

To help clarify these issues, as reported herein, in the same subset (n = 104) of participants in the recent, third adenoma recurrence trial noted above [14], we assessed the effects of supplemental calcium and vitamin D_3_, alone and in combination, over one year on markers of proliferation, differentiation, and apoptosis in the normal colorectal mucosa.

## Materials and methods

The participants in this adjunct biomarker study were recruited from participants in two of the 11 centers of a larger, randomized, placebo-controlled, partial 2×2 factorial chemoprevention clinical trial (n = 2,259) designed to test the efficacy of supplemental calcium and vitamin D_3_, alone and in combination, over 3–5 years on colorectal adenoma recurrence (NCT00153816) [14]. The design of the parent trial was previously reported [14]. Briefly, participants eligible for the trial were 45–75 years of age, in general good health, and had at least one histologically-verified neoplastic polyp ≥2 mm in diameter removed from the large bowel within 120 days of study entry. Exclusions from trial participation included invasive carcinoma, familial colonic polyposis syndromes or other serious intestinal disease, conditions that required or contraindicated supplemental calcium or vitamin D use, serum calcium concentrations outside the normal range, creatinine concentrations >20% above the upper limit of normal, and serum 25(OH)D concentrations <12 ng/ml (30 mmol/L or 0.00012 kg/m^3^) or >90 ng/ml (225 mmol/L or 0.00090 kg/m^3^). Additional exclusions from participation in the adjunct biomarker study included history of a bleeding disorder, current use of an anticoagulant medication, and inability to stop aspirin use for seven days.

Participants provided information on demographics, medical history, lifestyle, nutritional supplements, medications, and diet (using the Block Brief 2000 food frequency questionnaire [NutritionQuest, Berkeley, California]). Participants who adhered to the study protocol during the run-in period were then randomly assigned to a total of six treatment groups. The four main treatment groups were: placebo, supplemental calcium (1,200 mg of elemental calcium as calcium carbonate in equal divided doses twice daily), supplemental vitamin D_3_ (1,000 IU/day [25 μg/day], given as 500 IU [12.5 μg/day] twice daily), and both agents (“4-arm randomization”). Women who declined to forego calcium supplementation were randomized to calcium (1,200 mg/day) plus vitamin D_3_ (1,000 IU/day [25 μg/day]), or calcium 1,200 mg/day plus vitamin D placebo (“2-arm randomization”). Participants agreed to avoid taking calcium or vitamin D or supplements outside the trial; however, from April 2008 onwards, 1,000 IU (25 μg) of vitamin D and/or 400 mg or elemental calcium were permitted, though discouraged. Participants were randomized, stratified by clinical center, sex, follow-up colonoscopic examination at 3 or 5 years, and 4-arm or 2-arm randomization. Except for the statistician and data analyst, some programmers, and pharmacy personnel, all study staff and participants were blinded to treatment assignments.

During the treatment period, participants were interviewed via telephone every 6 months regarding their study treatment adherence, medication and supplement use, dietary calcium and vitamin D intake, colorectal procedures, and illnesses. Serum 25(OH)D, 1,25(OH)_2_D, calcium, and creatinine concentrations were measured at baseline and one year after randomization.

Funding for the adjunct biomarker study was received after the parent study began. For the adjunct biomarker study, 231 apparently eligible parent study participants at two clinical centers (Georgia and South Carolina) were offered participation near the end of the parent study’s pre-randomization, pill-taking run-in period, without knowledge of treatment assignment. The Institutional Review Board (IRB) for both clinical centers (the Emory IRB) approved the research (Emory IRB numbers IRB00012627 for the parent study [original approval February 16, 2004, and renewed annually] and IRB00000357 for the adjunct biomarker study [original approval October 14, 2005, and renewed annually]). A total of 109 participants met final eligibility criteria and provided written informed consent to participate. These participants underwent “non-prep” (i.e., no prior colon-cleansing preparations) biopsies of normal-appearing rectal mucosa at baseline and at a year-1 follow-up visit. A rigid sigmoidoscope and jumbo cup flexible biopsy forceps mounted on a semi-rigid rod were used to take six approximately 1 mm-thick biopsy specimens from the rectal mucosa 10 cm proximal to the external anal aperture. To avoid possible field effects, all biopsies were taken at least 4 cm from any polypoid lesions. Biopsies were teased onto a strip of bibulous paper and immediately placed in normal saline, oriented, and then transferred to 10% normal buffered formalin for 24 hours; they were then transferred to 70% ethanol, and, within a week, processed and embedded in paraffin blocks (two blocks of three biopsies per participant per biopsy visit). Sufficient biopsy tissue for biomarker measurements was obtained at baseline and 1-yr follow up on 104 patients.

### Immunohistochemistry protocol

The four biomarkers—mib-1, p21, bax, and bcl-2—were measured using automated immunohistochemistry and image analysis. First, to uncover epitopes, the slides were placed in a preheated Pretreatment Module (Lab Vision Corp., Fremount, CA) with 1× Citrate Buffer pH 6.0 (DAKO S1699, DAKO Corp., Carpinteria, CA [hereafter referred to as DAKO]) and steamed for 40 minutes. The slides were then placed in a DakoCytomation Autostainer Plus System (DAKO) automated immunostainer and immunohistochemically processed using a labeled streptavidin-biotin method (LSAB2 Detection System [DAKO K0675]) for mib-1, p21, and bcl-2, and a polymer system (Envision Plus Rabbit System [DAKO K4003]) for bax.

Commercially-available antibodies were selected based on previous literature and comparing the staining patterns found in positive and negative control tissues and in normal colorectal mucosa with those reported in the literature in which antibody specificity was validated. Information on the antibodies is as follows. For mib-1: antibody ID—AB_2142367; antibody name—Ki-67 antibody; clone ID—mib-1; clonality—monoclonal; target antigen—human recombinant peptide corresponding to a 1002 bp Ki-67 cDNA fragment; vendor/catalog number—DAKO M7240; and host organism—mouse. For p21: antibody ID—AB_2077700; antibody name—p21^WAF1/Cip1^ antibody; clone ID—SX118; clonality—monoclonal; target antigen—CDKN1A human; vendor/catalog number—DAKO M7202; and host organism—mouse. For bcl-2: antibody ID—AB_626733; antibody name—Bcl-2 (100) antibody; clone ID– 100; clonality—monoclonal; target antigen—human bcl-2; vendor/catalog number—Santa Cruz Biotechnology sc-509; and host organism—mouse. For bax: antibody name—rabbit anti-human bax antibody; clonality—polyclonal; target antigen—synthetic peptide corresponding to amino acids 43–61 of the human bax protein; vendor/catalog number—DAKO A3533; and host organism—rabbit. The antibodies were used in the following concentrations: mib-1–1:350; p21–1:40; bax– 1:200; and bcl-2–1:100).

The baseline and follow-up biopsy slides from each given participant were included in the same staining batch, and each batch included slide sets from a balance of participants from each treatment group, along with positive and negative control slides. A Leica CV5000 Coverslipper (Leica Microsystems, Inc., Buffalo Grove, IL) was used to coverslip the slides, which were not counterstained.

### Quantifying staining density of immunohistochemically detected biomarkers in colon crypts protocol (“scoring”)

**Fig 1:**
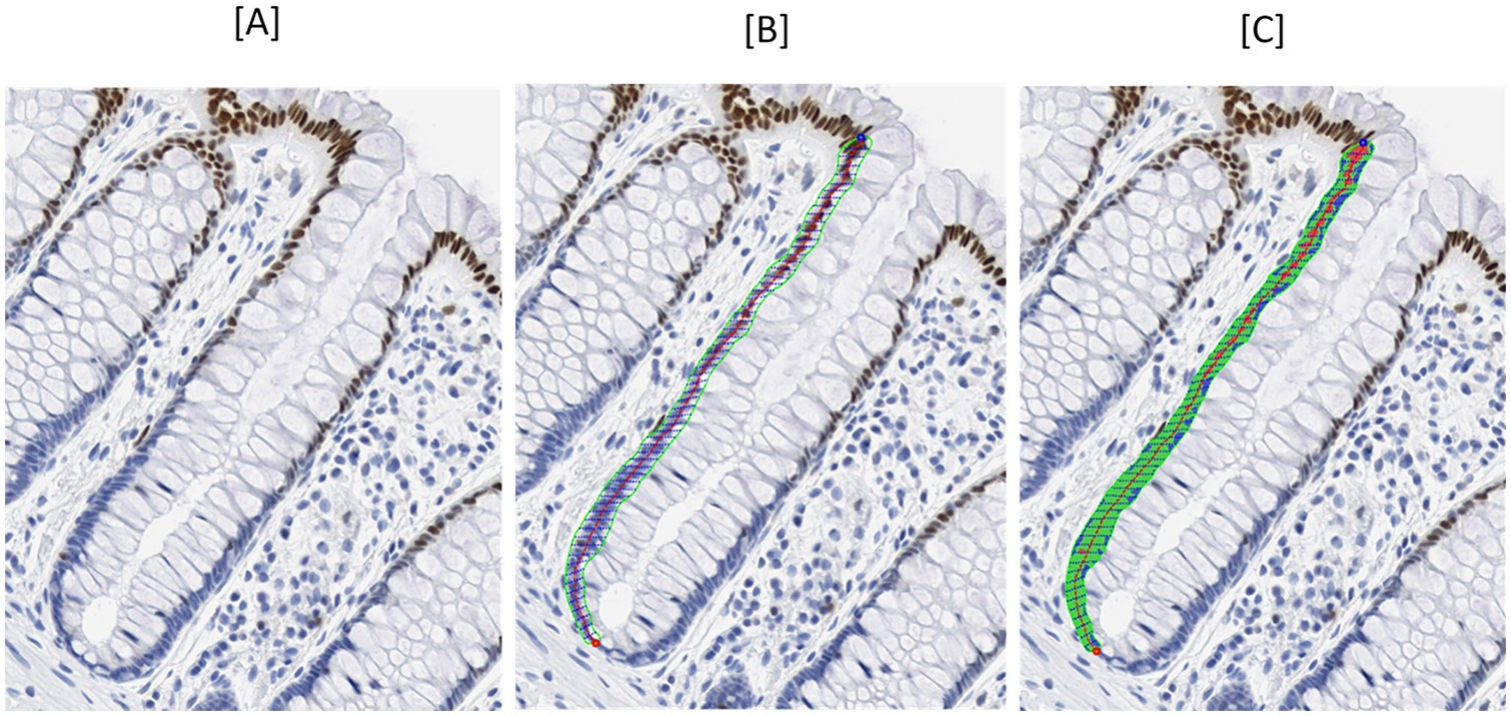
Depicts measurement of biomarker (p21) expression in crypts of normal appearing rectal mucosa using custom-designed quantitative image analysis software. [A] A full length hemicrypt is identified. [B] The hemicrypt is manually outlined, and the program automatically generates 50 segments. [C] p21 labeling optical density is quantified, overall and within each of the 50 segments of the hemicrypt.

A quantitative image analysis procedure (“scoring”) was used to describe and measure the detected biomarkers in colon crypts (Fig 1). The unit of analysis was a “hemicrypt,” defined as one-half of a longitudinally bisected crypt. A crypt defined as “scorable” was one that extended from the muscularis mucosae to the colon lumen.

The major equipment and software used for the image analysis procedures were: Scanscope CS Digital scanner (Aperio Technologies, Inc., CA), computer, digital drawing board, MatLab Software (MathWorks, Inc., MA), CellularEyes Image Analysis Suite (DivEyes LLC, GA), and MySQL (Sun Microsystems Inc., Santa Clara, CA). Throughout the scoring procedures, standardized settings were used for all equipment, and negative and positive control slides were checked for staining adequacy before analysis.

First, digital images of the slides were acquired using the Aperio Scanscope CS digital scanner, and then the CellularEyes program was used to review the electronic images and identify colon crypts acceptable for analysis. The technician was blinded to treatment assignments and used a strict protocol to select “scorable” hemicrypts for analysis as previously reported [15]. The borders of each selected hemicrypt were traced using the digital drawing board, and the program divided the outline into 50 equal-width segments of approximately the average width of normal colonocytes. The program then measured the background-corrected optical density of the biomarker labeling across the entire hemicrypt and within each segment. The resulting data were transferred automatically into the MySQL database. The previously described steps were then repeated for each subsequently identified hemicrypt, with a goal of scoring 32 hemicrypts. Subsamples of blinded slides were re-scored at intervals and after completing scoring to assess scoring reliability; the intra-class correlation coefficients for scoring reliability were >0.90 for each biomarker.

### Statistical analysis

The treatment groups were assessed for comparability of participant characteristics at baseline using the Fisher’s exact test for categorical variables and ANOVA or the Student t-test for continuous variables.

Because of the small sample sizes in some of the treatment groups—especially the double-placebo group (n = 12)—we combined treatment groups such that our main analyses were to assess changes in the expression of the biomarkers (individually and in relation to one another) from baseline to follow-up in the treatment groups that received (i) vitamin D relative to those that did not (“vitamin D vs. no vitamin D”), (ii) calcium relative to those that did not (“calcium vs. no calcium”), and (iii) calcium plus vitamin D relative to those that received calcium only (“calcium + vitamin D vs. calcium”). Because participants in the 2-arm randomization were not randomized to calcium, they were not included in the calcium analysis. We evaluated changes in the whole crypts as well as within crypt functional zones, including the upper 40% of crypts (the canonical differentiation zone), the lower 60% of the crypts (the canonical proliferation zone), and the ratio of the upper 40% of the crypts to the whole crypt (Φ_h_) [15]. The following ratios were calculated: bax/bcl-2, to represent the balance of pro- to anti-apoptosis factors (thus possibly better representing apoptosis); bax/mib-1, to represent the balance of apoptosis to proliferation; and p21/mib-1, to represent the balance of differentiation to proliferation. Each ratio was assessed for the whole crypt and the upper 40% and lower 60% of the crypts. All biomarker data were transformed by the natural logarithm to improve normality prior to hypothesis testing, and expressed as geometric means.

General mixed linear models were used to estimate treatment effects (differences in the expression of each biomarker variable from baseline to final follow-up) between participants in the treatment group of interest relative to those in the respective reference group. The base model included visit, treatment group, and a treatment group by visit interaction term. Also, because there were imbalances across the treatment groups on certain characteristics at baseline, we assessed those characteristics as potential confounders. These included sex, education level, non-steroidal anti-inflammatory drug use, current smoking status, multivitamin use, body mass index, total physical activity measured as metabolic equivalent of task (MET)-minutes, and total energy, total fat, dietary fiber, and total fruit and vegetable intakes. Potential treatment effect confounding was assessed through observing whether inclusion/exclusion of these covariates, individually or in combination, affected the estimated treatment effects by ≥10%; of the potential confounders, only total energy intake was retained in the final models.

Absolute treatment effects were calculated as: ([treatment follow-up—treatment baseline]–[reference group follow-up—reference group baseline]). Since the biomarker measurements were in optical density, to provide perspective on the magnitudes of the estimated treatment effects, relative effects ([treatment group follow-up / treatment group baseline] / [reference group follow-up / reference group baseline]) were taken from the model. Relative effects are interpreted similar to odds ratios (e.g., a relative effect of 1.2 would indicate that a biomarker increased 20% more in the treatment group relative to the control group).

Participants were retained in their originally assigned treatment group, regardless of adherence to study treatment and procedures. All statistical analyses were conducted using SAS 9.4 statistical software and SAS University Edition statistical software (SAS Institute Inc., Cary, NC). A two-sided P-value <0.05 was considered statistically significant.

## Results

Selected baseline characteristics of the study population are presented in Table 1. Among all participants, 46% were men, 79% were white, and the mean age was 58.9 years. The mean serum 25(OH)D concentration was 24.1 ± SD 9.3 ng/mL (60.25 ± SD 23.25 mmol/L or 0.000241 ± SD 0.000093 kg/m^3^). There were differences across the treatment groups in several participant characteristics as described three paragraphs above.

**Table 1:** Selected baseline characteristics of the adjunct biomarker study participants, according to treatment assignment (n = 104)^a^.

Characteristics	Treatment assignment							
	Randomization to vitamin D and to calcium (4-arm)					Randomization to vitamin D only (2-arm)		
	Placebo (n = 12)	Calcium (n = 16)	Vitamin D (n = 17)	Calcium + Vitamin D (n = 17)	*P*^b^	Placebo (n = 23)	Vitamin D (n = 19)	*P*^c^
***Demographics*, *medical history*, *habits*, *anthropometrics***								
Age, years	59.9 (7.2)	59.9 (6.5)	59.2 (7.8)	57.7 (7.1)	0.79	58.2 (5.3)	59.2 (7.3)	0.60
Male (%)	75	81	71	82	0.87	0	0	--
White (%)	83	75	71	94	0.42	70	84	0.57
≥ High school (%)	92	63	88	82	0.24	91	74	0.21
Take non-aspirin NSAID regularly (%)^d^	33	44	24	29	0.68	26	32	0.74
Current smoker (%)	25	6	0	6	0.10	0	16	0.16
Alcohol intake, drinks/day	0.7 (0.7)	0.8 (1.0)	0.9 (0.9)	0.9 (0.9)	0.92	0.5 (1.0)	0.3 (0.5)	0.40
Take multivitamin (%)	42	81	47	65	0.11	70	89	0.15
Physical activity, MET-mins/wk^e^	1,620 (1,195)	2,128 (2,378)	2,782 (2,784)	3,875 (2,424)	0.06	1,458 (1,235)	3,021 (3,469)	0.05
BMI, kg/m^2^	29.4 (4.9)	32.3 (7.6)	28.7 (5.5)	30.0 (4.5)	0.32	29.7 (5.6)	27.5 (4.7)	0.18
***Dietary intakes***								
Total energy intake, kcal/d	1,341 (358)	1,731 (537)	1,437 (527).	1,613 (550)	0.17	1,254 (549)	1,429 (595)	0.33
Total fat, g/d	57.6 (20.4)	68.8 (24.8)	60.5 (27.3)	62.6 (27.2)	0.68	50.3 (25.9)	61.5 (36.1)	0.25
Dietary fiber, g/d	10.4 (4.2)	15.6 (5.5)	13.7 (6.2)	15.6 (5.5)	0.05	13.8 (5.4)	17.2 (5.0)	0.04
Total fruits and vegetables, servings/day	3.3 (1.7)	4.4 (1.9)	4.5 (2.5)	4.4 (1.7)	0.35	4.7 (1.7)	6.0 (2.4)	0.04
Total calcium,^f^ mg/d	695.9 (414.7)	890.9 (255.4)	662.7 (271.8)	667.1 (254.7)	0.10	937.6 (466.5)	1,213 (554.3)	0.08
***Serum concentrations***								
25-OH-vitamin D, ng/ml^g^	22.4 (8.2)	24.5 (13.4)	23.1 (8.7)	22.5 (6.5)	0.93	24.8 (8.9)	26.5 (9.6)	0.54

Abbreviations: BMI, body mass index; MET, metabolic equivalent of task; NSAID, nonsteroidal anti-inflammatory drug

^a^ Data are given as means (SD) unless otherwise specified

^b^ By Fisher’s Exact test for categorical variables, and ANOVA for continuous variables

^c^ By Fisher’s Exact test for categorical variables, and t-test for continuous variables

^d^ At least once a week

^e^ One missing value in the vitamin D group, 2-arm

^f^ Dietary plus supplemental calcium intake

^g^ mmol/L = ng/ml x 2.5

Among the adjunct biomarker study participants, 76% reported taking ≥80% of their assigned study tablets during the one-year treatment period. The mean serum 25(OH)D increased from baseline to 1-year follow-up by 9.17 ± SD 9.68 ng/mL (22.97 ± SD 24.20 mmol/L or 0.0000917 ± SD 0.0000968 kg/m^3^) among participants assigned to the vitamin D treatment groups, and decreased by 1.69 ± SD 9.45 ng/mL (4.22 ± SD 23.62 mmol/L or 0.0000169 ± SD 0.0000945 kg/m^3^) among those who received vitamin D placebo.

The estimated treatment effects of vitamin D_3_ and calcium, separately and combined, on the biomarkers of proliferation and differentiation and of apoptosis are presented in Tables 2 and 3, respectively. While none of the findings was statistically significant, there were notable estimated changes in biomarker expression, as described below.

**Table 2:** Changes in biomarkers of proliferation and differentiation in colorectal crypts of the adjunct biomarker study participants (n = 104)^a^.

	Baseline			1-Year follow-up			Treatment effect			
Treatment group	n	Mean	95% CI	n	Mean	95% CI	Relative^b^	95% CI	*P*^c^	Abs^d^
**mib-1 (OD)**										
*Whole crypts*										
No vitamin D	51	1153	1032, 1289	51	1269	1135, 1418				
Vitamin D	52	1196	1072, 1335	53	1273	1141, 1419	0.97	0.82, 1.14	0.69	-39.3
No calcium	29	1315	1127, 1534	29	1259	1079, 1469				
Calcium	32	1216	1051, 1408	33	1236	1069, 1427	1.06	0.85, 1.32	0.59	74.8
Calcium alone	39	1068	942, 1210	39	1236	1091, 1401				
Vitamin D + calcium	35	1185	1039, 1352	36	1314	1154, 1497	0.96	0.80, 1.15	0.64	-39.8
*Upper 40% of crypts*										
No vitamin D	51	51.3	38.3, 68.8	51	55.3	41.3, 74.1				
Vitamin D	52	43.2	32.3, 57.6	53	38.9	29.2, 51.8	0.84	0.51, 1.36	0.47	-8.3
No calcium	29	55.1	36.5, 83.4	29	41.0	27.1, 62.0				
Calcium	32	43.8	29.6, 64.9	33	42.2	28.7, 62.1	1.29	0.65, 2.59	0.46	12.5
Calcium alone	39	44.6	32.4, 61.5	39	55.8	40.5, 76.9				
Vitamin D + calcium	35	43.9	31.3, 61.6	36	41.5	29.7, 58.0	0.76	0.45, 1.27	0.28	-13.6
*ϕh*										
No vitamin D	51	0.045	0.035, 0.057	51	0.044	0.034, 0.056				
Vitamin D	52	0.036	0.028, 0.046	53	0.031	0.024, 0.039	0.86	0.58, 1.28	0.46	0.004
No calcium	29	0.042	0.030, 0.059	29	0.033	0.023, 0.046				
Calcium	32	0.036	0.026, 0.050	33	0.034	0.025, 0.047	1.22	0.69, 2.15	0.49	0.007
Calcium alone	39	0.042	0.032, 0.055	39	0.045	0.034, 0.059				
Vitamin D + calcium	35	0.037	0.028, 0.049	36	0.032	0.024, 0.042	0.79	0.51, 1.21	0.28	0.008
**p21 (OD)**										
*Whole crypts*										
No vitamin D	51	599.4	507.5, 708.1	51	495.1	419.1, 584.8				
Vitamin D	53	603.2	512.2, 710.2	53	540.4	458.9, 636.3	1.08	0.86, 1.36	0.48	41.5
No calcium	29	631.1	519.9, 766.2	29	491.9	405.2, 597.1				
Calcium	33	652.3	544.1, 782.0	33	585.9	488.7, 702.5	1.15	0.89, 1.48	0.27	72.8
Calcium alone	39	580.0	478.7, 702.6	39	464.4	383.3, 562.6				
Vitamin D + calcium	36	580.1	475.1, 708.3	36	585.2	479.3, 714.6	1.26	0.96, 1.65	0.09	120.7
*Upper 40% of crypts*										
No vitamin D	51	318.9	265.8, 382.7	51	253.1	211.0, 303.7				
Vitamin D	53	316.9	265.0, 378.9	53	281.4	235.3, 336.4	1.12	0.88, 1.42	0.35	30.3
No calcium	29	344.7	277.7, 427.8	29	248.6	200.3, 308.6				
Calcium	33	357.5	292.0, 437.6	33	317.0	259.0, 388.1	1.23	0.93, 1.63	0.15	55.6
Calcium alone	39	302.5	245.4, 372.9	39	238.4	193.4, 293.9				
Vitamin D + calcium	36	303.8	244.3, 377.7	36	309.3	248.8, 384.6	1.29	0.98, 1.70	0.06	69.6
**p21/mib-1 (OD)**										
*Whole crypts*										
No vitamin D	51	0.52	0.45, 0.60	51	0.39	0.34, 0.45				
Vitamin D	53	0.50	0.44, 0.58	53	0.42	0.37, 0.49	1.13	0.90, 1.42	0.30	0.05
No calcium	29	0.50	0.42, 0.59	29	0.40	0.34, 0.48				
Calcium	33	0.54	0.47, 0.61	33	0.42	0.37, 0.47	0.95	0.74, 1.23	0.72	-0.02
Calcium alone	39	0.54	0.46, 0.64	39	0.38	0.32, 0.45				
Vitamin D + calcium	36	0.49	0.41, 0.58	36	0.45	0.37, 0.53	1.33	1.00, 1.75	0.05	0.12
*Upper 40% of crypts*										
No vitamin D	51	6.21	4.51, 8.56	51	4.57	3.32, 6.31				
Vitamin D	53	7.25	5.29, 9.93	53	7.24	5.29, 9.92	1.36	0.82, 2.26	0.24	1.63
No calcium	29	6.54	4.27, 10.02	29	6.34	4.14, 9.72				
Calcium	33	6.76	4.97, 9.19	33	5.33	3.92, 7.24	0.81	0.44, 1.51	0.51	-1.23
Calcium alone	39	6.78	4.72, 9.72	39	4.27	2.98, 6.13				
Vitamin D + calcium	36	6.81	4.68, 9.92	36	7.45	5.12, 10.85	1.73	0.98, 3.06	0.06	3.15

Abbreviations: Abs, absolute treatment effect; OD, optical density; 95% CI, 95% confidence interval; ϕ_h_, ratio of proliferation in upper 40% of crypt to whole crypt

^a^ Presented as geometric means and 95% confidence intervals.

^b^ Relative treatment effect from SAS Institute's Mixed Procedure defined as [(active treatment group follow-up mean) / (active treatment group baseline mean)] / [(reference group follow-up mean) / (reference group baseline mean)].

^c^ *P* value for difference between each active treatment group and reference group from repeated-measures MIXED model

^d^ Absolute treatment effect calculated as [(active treatment group follow-up mean)—(active treatment group baseline mean)]—[(reference group follow-up mean)—(reference group baseline mean)]

**Table 3:** Changes in biomarkers of apoptosis in colorectal crypts of the adjunct biomarker study participants (n = 104)^a^.

Treatment group	Baseline			1-Year follow-up			Treatment effect			
	n	Mean	95% CI	n	Mean	95% CI	Relative^b^	95% CI	*P*^c^	Abs^d^
**bax (OD)**										
*Whole crypts*										
No vitamin D	51	275.6	218.4, 347.8	51	381.9	302.6, 482.0				
Vitamin D	53	296.2	235.8, 372.3	53	397.3	316.2, 499.2	0.97	0.68, 1.38	0.86	-5.2
No calcium	29	289.3	208.8, 400.9	29	409.9	295.8, 567.9				
Calcium	33	295.1	217.5, 400.4	33	345.6	254.7, 468.9	0.83	0.52, 1.32	0.42	-70.1
Calcium alone	39	292.6	222.1, 385.5	39	350.9	266.4, 462.3				
Vitamin D + calcium	36	270.7	203.2, 360.6	36	410.7	308.2, 547.2	1.27	0.83, 1.92	0.26	81.8
**bcl-2 (OD)**										
*Whole crypts*										
No vitamin D	51	669.8	573.9, 781.7	51	706.7	605.5, 824.8				
Vitamin D	52	694.4	595.9, 809.3	52	752.4	645.7, 876.7	1.03	0.81, 1.30	0.82	21.1
No calcium	29	631.8	516.7, 772.6	29	693.6	567.2, 848.2				
Calcium	32	724.8	598.7, 877.5	32	701.7	579.6, 849.5	0.88	0.68, 1.15	0.34	-84.9
Calcium alone	39	671.8	560.7, 804.9	39	688.1	574.3, 824.5				
Vitamin D + calcium	35	717.5	592.8, 868.4	35	787.5	650.6, 953.1	1.07	0.79, 1.44	0.65	53.7
**bax/bcl-2 (OD)**										
*Whole crypts*										
No vitamin D	51	0.41	0.33, 0.52	51	0.54	0.43, 0.68				
Vitamin D	52	0.44	0.35, 0.55	52	0.53	0.42, 0.66	0.92	0.66, 1.29	0.64	-0.04
No calcium	29	0.45	0.33, 0.62	29	0.58	0.43, 0.80				
Calcium	32	0.46	0.36, 0.57	32	0.53	0.42, 0.66	0.90	0.60, 1.34	0.59	-0.06
Calcium alone	39	0.44	0.33, 0.57	39	0.51	0.39, 0.66				
Vitamin D + calcium	35	0.39	0.29, 0.51	35	0.52	0.39, 0.69	1.15	0.79, 1.68	0.47	0.06
**bax/mib-1 (OD)**										
*Whole crypts*										
No vitamin D	51	0.24	0.19, 0.30	51	0.30	0.24, 0.38				
Vitamin D	53	0.25	0.19, 0.31	53	0.31	0.25, 0.40	1.01	0.68, 1.48	0.98	0.00
No calcium	29	0.22	0.16, 0.31	29	0.33	0.24, 0.46				
Calcium	33	0.26	0.21, 0.33	33	0.29	0.23, 0.36	0.75	0.47, 1.18	0.21	-0.08
Calcium alone	39	0.27	0.21, 0.36	39	0.28	0.22, 0.37				
Vitamin D + calcium	36	0.23	0.17, 0.30	36	0.31	0.23, 0.42	1.33	0.85, 2.08	0.21	0.07
*Upper 40% of crypts*										
No vitamin D	51	1.34	0.89, 2.01	51	2.01	1.34, 3.02				
Vitamin D	53	1.87	1.26, 2.79	53	2.94	1.98, 4.37	1.04	0.54, 2.01	0.90	0.40
No calcium	29	1.36	0.79, 2.34	29	2.97	1.72, 5.13				
Calcium	33	1.60	1.08, 2.37	33	2.07	1.40, 3.07	0.59	0.26, 1.33	0.20	-1.14
Calcium alone	39	1.73	1.10, 2.72	39	1.86	1.18, 2.92				
Vitamin D + calcium	36	1.65	1.03, 2.64	36	2.80	1.75, 4.48	1.58	0.77, 3.24	0.21	1.02

Abbreviations: Abs, absolute treatment effect; OD, optical density; 95% CI, 95% confidence

Interval

^a^ Presented as geometric means and 95% confidence intervals.

^b^ Relative treatment effect from SAS Institute's Mixed Procedure defined as [(active treatment group follow-up mean) / (active treatment group baseline mean)] / [(reference group follow-up mean) / (reference group baseline mean)].

^c^ *P* value for difference between each active treatment group and reference group from

^d^ Absolute treatment effect calculated as [(active treatment group follow-up mean)—(active treatment group baseline mean)]—[(reference group follow-up mean)—(reference group baseline mean)

### Proliferation

As noted in Table 2, in the vitamin D alone and the vitamin D + calcium groups relative to their respective reference groups, mib-1 expression decreased 16% and 24%, respectively, in the upper 40% of crypts (crypt differentiation zone), and 14% and 21% in the ϕ_h_ of crypts. In contrast, in the calcium relative to the no calcium group, mib-1 expression increased 29% and 22% in the upper 40% and the ϕ_h_ of crypts, respectively. All of the estimated treatment effects on mib-1 expression in the whole (Table 2) and lower 60% (proliferation zone) of crypts (S1 Table) were close to the null.

### Differentiation

p21 expression was almost exclusively in the upper 40% of crypts, where it was estimated to increase in all treatment groups relative to their respective reference groups (Table 2). In the vitamin D + calcium, calcium alone, and vitamin D alone groups, the estimated increases were 29% (nearly statistically significant), 23%, and 12% (the findings for the whole crypt were similar to these, but of minimally less magnitude). The findings for the p21/mib-1 ratio were a 73% (nearly statistically significant) increase in the vitamin D + calcium group, a 19% decrease in the calcium group, and a 36% increase in the vitamin D alone group (the findings for the whole crypt were of similar, but lesser magnitude).

### Apoptosis

As noted in Table 3, among the treatment-groups-of-interest relative to their respective reference groups, bax expression in the whole crypt was estimated to increase only in the vitamin D + calcium group (an estimated increase of 27%), whereas the estimated changes in the other treatment groups were close to the null. The findings for the upper 40% and 60% of crypts were similar to those for the whole crypt (S2 Table). For all treatment groups, the estimated treatment effects on bcl-2 expression in the whole crypt (Table 3) and the crypt sub-zones (S2 Table) were close to the null. The findings for the bax/bcl-2 ratio, were similar to, but of lesser magnitude than, those for bax alone. The findings for the bax/mib-1 ratio were increases of 58% and 33%, respectively, in the upper 40% of crypts and the whole crypt in the vitamin D + calcium group, whereas the findings for the vitamin D alone group were close to the null, and the corresponding findings for the calcium alone group were for estimated decreases of 41% and 25%.

## Discussion

Our findings in this pilot, adjunct biomarker study suggest that, in sporadic colorectal adenoma patients, daily vitamin D_3_ supplementation, alone or—especially—in combination with calcium, may shift downwards (“normalize”) the distribution of proliferating cells in colorectal crypts, and increase colorectal epithelial cell differentiation and apoptosis, especially in relation to proliferation. Our findings also suggest that, consistent with our hypotheses and previous findings, calcium may increase differentiation; however, in contrast with our hypotheses and previous findings, our findings for calcium were consistent with it modestly increasing proliferation and decreasing apoptosis.

As noted in the Introduction and reviewed extensively elsewhere, the potential efficacy of vitamin D and calcium as chemopreventive agents against colorectal cancer is supported by strong biological plausibility and animal experimental and human evidence [3–5]. Calcium intakes and circulating 25(OH)D concentrations have been consistently associated with lower risk for colorectal neoplasms [3–5], and calcium supplementation reduced colorectal adenoma recurrence in two large, randomized controlled trials [9, 10]. However, in the large randomized, controlled trial (n = 2,259) in which the present study was nested, there was no overall effect of calcium 1,200 mg/day and/or vitamin D_3_ 1,000 IU/day (25 μg/day) over 3–5 years on colorectal adenoma recurrence [14].

Calcium and vitamin D are thought to protect against colorectal carcinogenesis via multiple mechanisms. If the amount of calcium consumed exceeds that which binds with phosphate in the gut lumen and is absorbed for maintaining calcium homeostasis, the remaining free calcium can bind the bile acids and fatty acids resulting from fat digestion, thus protecting colonocytes from oxidative damage to DNA [4] and to cell structures, thus reducing compensatory hyperproliferation and inflammatory responses [6]. Also, *in vitro*, calcium reduces cell proliferation and increases cell differentiation; these effects may be mediated via calcium binding with the calcium sensing receptor, which downregulates pro-proliferative β-catenin and upregulates the cell adhesion molecule E-cadherin [4, 7, 8]. Vitamin D binds with the vitamin D receptor (which is abundantly expressed in the colorectal mucosa), which upregulates CYP3A4, which in turn catabolizes the particularly toxic secondary bile acid lithocholic acid [3, 7]. Vitamin D also modulates >200 genes involved in, among others, bile acid metabolism, cell cycle regulation, cell adhesion, and growth factor signaling [3, 7]. Thus, vitamin D and calcium may, at least in part, reduce risk via indirectly and directly modulating the cell cycle.

The cell cycle biomarkers used in our study are well recognized in the literature. Ki-67 (reliably detected via an antibody to its mib-1 epitope), is a non-histone protein located in the nucleus of proliferating, but not quiescent G_0_ phase, cells [16]. p21^waf1/cip1^, a cyclin-dependent kinase inhibitor, has important roles in cell cycle regulation, and p21 expression downregulation is a common occurrence in colorectal cancer [17]. Bax promotes apoptosis by breaching mitochondrial outer membrane integrity [18], while bcl-2 inhibits bax activity, therefore inhibiting apoptosis [19]. The bax/bcl-2 ratio, representing the proportion of apoptosis promotion to inhibition, may be a positive prognostic marker in colorectal cancer patients [20]. Evidence from *in vitro* [21–23] and animal [24, 25] studies suggest that calcium and vitamin D may enhance colonocyte differentiation. Calcium and vitamin D are involved in the activation of the calcium-sensing receptor and protein kinase C, which activates various downstream pathways that may modulate colonocyte differentiation and apoptosis [7, 26, 27].

The effects of vitamin D and calcium, separately and combined, on cell cycle markers in the crypts of the normal-appearing rectal mucosa of sporadic colorectal adenoma patients were estimated in a previous pilot, 2x2 factorial design chemoprevention trial (n = 92) (the Calcium and Vitamin D vs. Markers of Adenomatous Polyps trial, or CaD v MAP) [11, 12], and the efficacy of calcium alone in modulating proliferation was tested in a previous full-scale trial (n = 193) in adenoma patients (the Calcium and Colorectal Epithelial Cell Proliferation trial, or CCECP) [13]. A comparison of findings from the present trial with those from the two previous trials is presented in Table 4, and summarized and discussed below.

**Table 4:** Comparisons of selected findings^a^ in three randomized, double-blind, placebo-controlled chemoprevention trials with cell cycle marker endpoints^b^.

Biomarker	Crypt Parameter	Trials							
		PPS4B^c^			CaD v MAP^d^			CCECP^e^	
		Calcium (1.2 g/d)	Vitamin D_3_ (1,000 IU/d)	Ca^++^ + Vit. D^f^	Calcium (2.0 g)	Vitamin D_3_ (800 IU/d)	Ca^++^ + Vit. D^g^	Calcium (1.0 g/d)	Calcium (2.0 g/d)
**Proliferation**^h^	Whole crypt	↑ 6%	↓3%	↓ 4%	↑ 23%	↑ 32%	↓ 11%	↓ 8%	↓ 9%
	ϕ_h_	↑ 22%	↓ 14%	↓ 21%	↓ 6%	↓ 3%	↓ 3%	↓ 59%	↓ 69%^i^
**p21**	Whole crypt	↑ 15%	↑ 8%	↑ 26%	↑ 101%^i^	↑ 142%^i^	↑ 25%	-	-
**p21/mib-1**	Whole crypt	↓ 5%	↑ 13%	↑ 33%^i^	-	-	-	-	-
**bax**	Whole crypt	↓ 17%	↓ 3%	↑ 27%	↑ 33%	↑ 56%^i^	↑ 33%	-	-
**bax/mib-1**	Whole crypt	↓ 25%	↑ 1%	↑ 33%	-	-	-	-	-
**bcl-2**	Whole crypt	↓ 12%	↑ 3%	↑ 7%	↓ 20%	↑ 14%	↓ 16%	-	-
**bax/bcl-2**	Whole crypt	↓ 10%	↓ 8%	↑ 15%	↑ 62%	↑ 47%	↑ 71%	-	-

Abbreviations: PPS4B, Polyp Prevention Study 4 Biomarkers study; CaD v MAP, Calcium and Vitamin D vs. Markers of Adenomatous Polyps trial; CCECP, Calcium and Colorectal Epithelial Cell Proliferation trial, Ca++, calcium; Vit. D, vitamin D_3_; ϕ_h_, ratio of proliferation in upper 40% of crypt to whole crypt

^a^ Proportional changes in the active relative to the control group

^b^ Cell cycle markers measured in crypts in biopsies of normal-appearing rectal mucosa via immunohistochemistry and image analysis

^c^ The present study, n = 104, intervention duration = 12 months

^d^ n = 92, intervention duration = 6 months [11, 12]

^e^ n = 193, intervention duration = 6 months [13]

^f^ Both calcium 1.2 g/d and vitamin D_3_ 1,000 IU/d (25 μg/day)

^g^ Both calcium 2.0 g/d and vitamin D_3_ 800 IU/d (20 μg/day)

^h^ Measured via detection of mib-1 in PPS4B and CaD v MAP, and PCNA (proliferating cell nuclear antigen) in CCECP

^i^ p < 0.05

### Proliferation

Across the three trials, the estimated treatment effects of calcium, vitamin D, and calcium plus vitamin D on markers of proliferation in the whole crypt, were, for the most part, variable and close to the null, and none was statistically significant; however, in all treatment groups (except the calcium only group in the present study) there was an estimated decrease in the ϕ_h_ of crypts, a finding that was statistically significant for the 2.0 g calcium group (and, not shown, the merged 1.0 g and 2.0 g calcium groups) in the CCECP trial.

Thus, none of the three trials supports that calcium substantially affects the overall proliferation rate, but the previous trials—especially the full-scale trial—support that calcium helps maintain the normal distribution of the proliferation zone. That the finding in the present study for the effect of calcium alone on the ϕ_h_ of crypts is not in line with those in the previous trials may be attributable to chance and other limitations of the present study as discussed further below. The results of the two factorial design trials also do not support a role for vitamin D in reducing the overall proliferation rate, but are consistent with vitamin D, alone or combined with calcium, in normalizing the proliferation zone.

### Differentiation

In the present study and the CaD v MAP trial, p21 expression was estimated to increase in every treatment group, and the findings for calcium and vitamin D alone were statistically significant in the latter trial. In the present trial, the findings for the p21/mib-1 ratio were not substantially different from those for p21 alone.

Thus, the findings from the present and CaD v MAP trials support that calcium and vitamin D alone or combined may promote colorectal crypt epithelial cell differentiation. Further investigation is needed into the p21/mib-1 ratio as an indicator of differentiation relative to proliferation in colorectal crypts.

### Apoptosis

In the CaD v MAP trial, there were estimated substantial increases in bax expression in all treatment groups, especially the vitamin D alone group, for which the finding was statistically significant, whereas in the present study, bax was estimated to increase only in the calcium + vitamin D group. In the present trial, the findings for the bax/mib-1 ratio were not substantially different from those for bax alone. Across the two pilot factorial design trials, the estimated effects of all active treatments on bcl-2 in the whole crypt, were, for the most part, variable and close to the null, and none was statistically significant; however, in the CaD v MAP trial, there were estimated substantial increases in the bax/bcl-2 ratio in all three active treatment groups—especially in the calcium + vitamin D group (an estimated 71% increase)—whereas in the present study, the findings were closer to the null, except for an estimated 15% increase in the calcium + vitamin D group.

Thus, the findings from the present and CaD v MAP trials suggest that, at least calcium and vitamin D combined may promote apoptosis in crypts in the normal-appearing colorectal mucosa of sporadic adenoma patients, but the two studies differed in the estimated effects of calcium and vitamin D alone, with the CaD v MAP trial suggesting substantial apoptosis-promoting effects, and the present trial suggesting essentially no effects. Possible reasons for the discrepant findings are discussed below. The results from the two studies do not support substantial calcium and/or vitamin D effects on bcl-2 expression, nor advantages of the bax/bcl-2 ratio over bax expression alone when using our measurement methodology. Further investigation of the effects of calcium and/or vitamin D on bax expression or on other biomarkers of apoptosis is needed.

As described above, the findings across the present and previous trials are mostly concordant, but there are some differences for which there are several possible reasons. The present and CaD v MAP studies had relatively small sample sizes, suggesting that chance may have played a role. The present study’s calcium dose was smaller than the CaD v MAP trial’s dose, suggesting that a larger dose may be more efficacious. Also, the present study had some unique limitations. Participants in the present study were recruited from the larger adenoma recurrence trial without knowledge of their treatment group assignments. Consequently, the sample sizes for the treatment groups were unequal, and the small sample sizes for some of the treatment groups necessitated combining groups for analysis, making for a less “clean” analysis. Also, the small double-placebo group (n = 12), perhaps due to chance, was particularly influential in our results, especially those for the calcium only group and for bax, both of which were the most discordant from our previous findings. Second, the treatment groups were not balanced on various participant characteristics, which, even though adjustment for them in our analyses did not materially affect our results, leaves the potential for treatment effect confounding by unmeasured factors. It is also possible that study population differences affected treatment responses. Support for this is that calcium reduced adenoma recurrence in two previous, large trials, but not in the trial in which the present study was nested.

Although the parent trial in which the present study was nested found no overall effects on adenoma recurrence, the present study’s findings are relevant. As noted above, the biological plausibility for calcium and vitamin D, the animal experimental evidence, the extensive human observational literature, and the two previous adenoma recurrence trials support that calcium and vitamin D may reduce risk for colorectal neoplasms [3–5, 28, 29]. In rodent models, increased proliferation in the normal-appearing colonic mucosa preceded tumor formation, and calcium supplementation ameliorated proliferation and reduced tumorigenesis [28]. In the same participants investigated in the present study, and in the CaD v MAP trial, we estimated that vitamin D_3_, alone or combined with calcium, modified APC, β-catenin, and E-cadherin (all three involved in the APC colon carcinogenesis pathway) expression in directions hypothesized to reduce risk for colorectal neoplasms [15]. Collectively, these results suggest that calcium and vitamin D may reduce risk for colorectal neoplasms, and that they do so by acting on the normal mucosa to prevent early stage colorectal carcinogenesis.

An issue with adenoma recurrence trials is that it is commonly thought that it takes many years to develop a macroscopic adenoma from a completely normal mucosa, and a substantial proportion of persons diagnosed with adenomas develop subsequent adenomas (in the parent trial population about 40% did). This indicates that a substantial proportion of persons who have adenomas removed may have fairly committed clones of cells in the “pipeline” to become macroscopic adenomas in the ensuing few years. So, if a preventive agent is primarily effective in preventing the very earliest stages of colon carcinogenesis, an effect on preventing recurrent adenomas may not be detectable until enough years (e.g., >5 years) elapse for the committed clones to have progressed to a detectable stage. The pattern of 3- vs. 5-year findings in the parent trial, and the long-term follow up of participants in the previous calcium and adenoma recurrence trial (stronger estimates 5 years post trial vs. at trial end [OR 0.63 vs. 0.85]) also are consistent with this [30].

While the present study had the above-noted limitations, it also had several strengths, including the double-blind, placebo-controlled design, high protocol adherence by study participants, automated immunohistochemistry, novel quantitative image analysis software, and high level of biomarker measurement reliability. Also, to our knowledge, this was the first human study to investigate the effects of vitamin D and calcium supplementation on differentiation and apoptosis relative to proliferation in the normal-appearing human colorectal mucosa.

### Conclusions

In summary, the results from this pilot clinical trial, taken together with previous literature, suggest that 1) calcium and vitamin D, alone or together, while they may not affect the overall proliferation rate, may normalize the distribution of the crypt proliferation zone and increase differentiation; and 2) that vitamin D combined with calcium may increase apoptosis in the normal human colorectal epithelium. Also, the collective findings do not support substantial calcium and/or vitamin D effects on bcl-2 expression, nor advantages of the bax/bcl-2 ratio over bax expression alone when using our measurement methodology. The collective findings also indicate that further investigation of the following is needed: the p21/mib-1 ratio as an indicator of differentiation relative proliferation in colorectal crypts, and the effects of calcium and/or vitamin D on the expression of bax or other biomarkers of apoptosis. Finally, based on our findings, literature review, and discussion, calcium and vitamin D remain viable chemopreventive agents against colorectal neoplasms, and mib-1, p21, and bax expression in the normal-appearing colorectal mucosa remain viable, treatable pre-neoplastic biomarkers of risk for colorectal neoplasms.

## Supporting information

S1 Table Changes in biomarkers of proliferation and differentiation in colorectal crypts of the adjunct biomarker study participants (n = 104). (DOCX)

S2 Table Changes in biomarkers of apoptosis in colorectal crypts of the adjunct biomarker study participants (n = 104). (DOCX)

S1 File Data for analyses. (XLSX)

## References

[1] American Cancer Society. Cancer Facts & Figures 2017. Atlanta: American Cancer Society; 2017 https://www.cancer.org/content/dam/cancer-org/research/cancer-facts-and-statistics/annual-cancer-facts-and-figures/2017/cancer-facts-and-figures-2017.pdf.

[2] American Cancer Society. Colorectal Cancer Facts & Figures 2017–2019. Atlanta: American Cancer Society; 2017 https://www.cancer.org/content/dam/cancer-org/research/cancer-facts-and-statistics/colorectal-cancer-facts-and-figures/colorectal-cancer-facts-and-figures-2017-2019.pdf.

[3] Bostick RM. Effects of supplemental vitamin D and calcium on normal colon tissue and circulating biomarkers of risk for colorectal neoplasms. J Steroid Biochem Mol Biol. 2015;148:86–95. 10.1016/j.jsbmb.2015.01.010 25597952 10.1016/j.jsbmb.2015.01.010 PMC4389892

[4] Song M, Garrett WS, Chan AT. Nutrients, foods, and colorectal cancer prevention. Gastroenterology. 2015;148(6):1244–60 e16. 10.1053/j.gastro.2014.12.035 25575572 10.1053/j.gastro.2014.12.035 PMC4409470

[5] Zhang X, Giovannucci E. Calcium, vitamin D and colorectal cancer chemoprevention. Best Pract Res Clin Gastroenterol. 2011;25(4–5):485–94. 10.1016/j.bpg.2011.10.001 22122765 10.1016/j.bpg.2011.10.001

[6] Newmark HL, Lipkin M. Calcium, vitamin D, and colon cancer. Cancer Res. 1992;52(7 Suppl):2067s–70s. 1544142

[7] Lamprecht SA, Lipkin M. Chemoprevention of colon cancer by calcium, vitamin D and folate: molecular mechanisms. Nat Rev Cancer. 2003;3(8):601–14. 10.1038/nrc1144 12894248 10.1038/nrc1144

[8] Peterlik M, Kallay E, Cross HS. Calcium nutrition and extracellular calcium sensing: relevance for the pathogenesis of osteoporosis, cancer and cardiovascular diseases. Nutrients. 2013;5(1):302–27. 10.3390/nu5010302 23340319 10.3390/nu5010302 PMC3571650

[9] Baron JA, Beach M, Mandel JS, van Stolk RU, Haile RW, Sandler RS, et al Calcium supplements for the prevention of colorectal adenomas. Calcium Polyp Prevention Study Group. N Engl J Med. 1999;340(2):101–7. 10.1056/NEJM199901143400204 9887161 10.1056/NEJM199901143400204

[10] Bonithon-Kopp C, Kronborg O, Giacosa A, Rath U, Faivre J. Calcium and fibre supplementation in prevention of colorectal adenoma recurrence: a randomised intervention trial. European Cancer Prevention Organisation Study Group. Lancet. 2000;356(9238):1300–6. 11073017 10.1016/s0140-6736(00)02813-0

[11] Fedirko V, Bostick RM, Flanders WD, Long Q, Shaukat A, Rutherford RE, et al Effects of vitamin D and calcium supplementation on markers of apoptosis in normal colon mucosa: a randomized, double-blind, placebo-controlled clinical trial. Cancer Prev Res. 2009;2(3):213–23. 10.1158/1940-6207.CAPR-08-0157 PMC2712935 19258546

[12] Fedirko V, Bostick RM, Flanders WD, Long Q, Sidelnikov E, Shaukat A, et al Effects of vitamin D and calcium on proliferation and differentiation in normal colon mucosa: a randomized clinical trial. Cancer Epidemiol Biomarkers Prev. 2009;18(11):2933–41. 10.1158/1055-9965.EPI-09-0239 19861511 10.1158/1055-9965.EPI-09-0239 PMC2784000

[13] Bostick RM, Fosdick L, Wood JR, Grambsch P, Grandits GA, Lillemoe TJ, et al Calcium and colorectal epithelial cell proliferation in sporadic adenoma patients: a randomized, double-blinded, placebo-controlled clinical trial. J Natl Cancer Inst. 1995;87(17):1307–15. 7658483 10.1093/jnci/87.17.1307

[14] Baron JA, Barry EL, Mott LA, Rees JR, Sandler RS, Snover DC, et al A trial of calcium and vitamin D for the prevention of colorectal adenomas. N Engl J Med. 2015;373(16):1519–30. 10.1056/NEJMoa1500409 26465985 10.1056/NEJMoa1500409 PMC4643064

[15] Liu S, Barry EL, Baron JA, Rutherford RE, Seabrook ME, Bostick RM. Effects of supplemental calcium and vitamin D on the APC/beta-catenin pathway in the normal colorectal mucosa of colorectal adenoma patients. Mol Carcinog. 2017;56(2):412–24. 10.1002/mc.22504 27254743 10.1002/mc.22504 PMC5586148

[16] Martin B, Paesmans M, Mascaux C, Berghmans T, Lothaire P, Meert AP, et al Ki-67 expression and patients survival in lung cancer: systematic review of the literature with meta-analysis. Br J Cancer. 2004;91(12):2018–25. 10.1038/sj.bjc.6602233 15545971 10.1038/sj.bjc.6602233 PMC2409786

[17] Waldman T, Lengauer C, Kinzler KW, Vogelstein B. Uncoupling of S phase and mitosis induced by anticancer agents in cells lacking p21. Nature. 1996;381(6584):713–6. 10.1038/381713a0 8649519 10.1038/381713a0

[18] Tait SW, Green DR. Mitochondria and cell death: outer membrane permeabilization and beyond. Nat Rev Mol Cell Biol. 2010;11(9):621–32. 10.1038/nrm2952 20683470 10.1038/nrm2952

[19] Murphy KM, Ranganathan V, Farnsworth ML, Kavallaris M, Lock RB. Bcl-2 inhibits bax translocation from cytosol to mitochondria during drug-induced apoptosis of human tumor cells. Cell Death Differ. 2000;7(1):102–11. 10.1038/sj.cdd.4400597 10713725 10.1038/sj.cdd.4400597

[20] Khodapasand E, Jafarzadeh N, Farrokhi F, Kamalidehghan B, Houshmand M. Is bax/bcl-2 ratio considered as a prognostic marker with age and tumor location in colorectal cancer? Iran Biomed J. 2015;19(2):69–75. 10.6091/ibj.1366.2015 25864810 10.6091/ibj.1366.2015 PMC4412916

[21] Aggarwal A, Hobaus J, Tennakoon S, Prinz-Wohlgenannt M, Graca J, Price SA, et al Active vitamin D potentiates the anti-neoplastic effects of calcium in the colon: a cross talk through the calcium-sensing receptor. J Steroid Biochem Mol Biol. 2016;155(Pt B):231–8. 10.1016/j.jsbmb.2015.02.006 25758239 10.1016/j.jsbmb.2015.02.006

[22] Di Rosa M, Malaguarnera M, Zanghi A, Passaniti A, Malaguarnera L. Vitamin D3 insufficiency and colorectal cancer. Crit Rev Oncol Hematol. 2013;88(3):594–612. 10.1016/j.critrevonc.2013.07.016 23941729 10.1016/j.critrevonc.2013.07.016

[23] Wranicz J, Szostak-Wegierek D. Health outcomes of vitamin D. Part II. Role in prevention of diseases. Rocz Panstw Zakl Hig. 2014;65(4):273–9. 25526571

[24] Hummel DM, Thiem U, Hobaus J, Mesteri I, Gober L, Stremnitzer C, et al Prevention of preneoplastic lesions by dietary vitamin D in a mouse model of colorectal carcinogenesis. J Steroid Biochem Mol Biol. 2013;136:284–8. 10.1016/j.jsbmb.2012.09.003 22982628 10.1016/j.jsbmb.2012.09.003 PMC3695567

[25] Nittke T, Kallay E, Manhardt T, Cross HS. Parallel elevation of colonic 1,25-dihydroxyvitamin D3 levels and apoptosis in female mice on a calcium-deficient diet. Anticancer Res. 2009;29(9):3727–32. 19667171

[26] Lamprecht SA, Lipkin M. Cellular mechanisms of calcium and vitamin D in the inhibition of colorectal carcinogenesis. Ann N Y Acad Sci. 2001;952:73–87. 11795445 10.1111/j.1749-6632.2001.tb02729.x

[27] Brenner BM, Russell N, Albrecht S, Davies RJ. The effect of dietary vitamin D3 on the intracellular calcium gradient in mammalian colonic crypts. Cancer Lett. 1998;127(1–2):43–53. 9619857 10.1016/s0304-3835(98)00005-6

[28] Beaty MM, Lee EY, Glauert HP. Influence of dietary calcium and vitamin D on colon epithelial cell proliferation and 1,2-dimethylhydrazine-induced colon carcinogenesis in rats fed high-fat diets. J Nutr. 1993;123(1):144–52. 10.1093/jn/123.1.144 8421225 10.1093/jn/123.1.144

[29] Kawaura A, Tanida N, Sawada K, Oda M, Shimoyama T. Supplemental administration of 1-alpha-hydroxyvitamin-D3 inhibits promotion by intrarectal instillation of lithocholic acid in N-methyl-N-nitrosourea-induced colonic tumorigenesis in rats. Carcinogenesis. 1989;10(4):647–9. 2702712 10.1093/carcin/10.4.647

[30] Grau MV, Baron JA, Sandler RS, Wallace K, Haile RW, Church TR, et al Prolonged effect of calcium supplementation on risk of colorectal adenomas in a randomized trial. J Natl Cancer Inst. 2007;99(2):129–36. 10.1093/jnci/djk016 17227996 10.1093/jnci/djk016

