# Supplementary material for: Effects of supplemental vitamin D and calcium on markers of proliferation, differentiation, and apoptosis in the normal colorectal mucosa of colorectal adenoma patients
Source: PLoS One. 2018 Dec 17;13(12):e0208762. doi: 10.1371/journal.pone.0208762 (PMC6296527; doi:10.1371/journal.pone.0208762)
Supplement: S1 Table — (DOCX) [file pone.0208762.s001.docx]

**Supplemental Table 1. Changes in biomarkers of proliferation and differentiation in colorectal crypts of the adjunct biomarker study participants (n = 104)^a^.**

|  | **Baseline** | | | **1-Year follow-up** | | | **Treatment effect** | | | |
| --- | --- | --- | --- | --- | --- | --- | --- | --- | --- | --- |
| **Treatment group** | **n** | **Mean** | **95% CI** | **n** | **Mean** | **95% CI** | **Relative**^b^ | **95% CI** | ***P****^c^* | **Abs**^d^ |
| **mib-1 (OD)** |  |  |  |  |  |  |  |  |  |  |
| *Whole crypts* |  |  |  |  |  |  |  |  |  |  |
| No vitamin D | 51 | 1153 | 1032, 1289 | 51 | 1269 | 1135, 1418 |  |  |  |  |
| Vitamin D | 52 | 1196 | 1072, 1335 | 53 | 1273 | 1141, 1419 | 0.97 | 0.82, 1.14 | 0.69 | -39.3 |
| No calcium | 29 | 1315 | 1127, 1534 | 29 | 1259 | 1079, 1469 |  |  |  |  |
| Calcium | 32 | 1216 | 1051, 1408 | 33 | 1236 | 1069, 1427 | 1.06 | 0.85, 1.32 | 0.59 | 74.8 |
| Calcium alone | 39 | 1068 | 942, 1210 | 39 | 1236 | 1091, 1401 |  |  |  |  |
| Vitamin D + calcium | 35 | 1185 | 1039, 1352 | 36 | 1314 | 1154, 1497 | 0.96 | 0.80, 1.15 | 0.64 | -39.8 |
|  |  |  |  |  |  |  |  |  |  |  |
| *Upper 40% of crypts* | |  |  |  |  |  |  |  |  |  |
| No vitamin D | 51 | 51.3 | 38.3, 68.8 | 51 | 55.3 | 41.3, 74.1 |  |  |  |  |
| Vitamin D | 52 | 43.2 | 32.3, 57.6 | 53 | 38.9 | 29.2, 51.8 | 0.84 | 0.51, 1.36 | 0.47 | -8.3 |
| No calcium | 29 | 55.1 | 36.5, 83.4 | 29 | 41.0 | 27.1, 62.0 |  |  |  |  |
| Calcium | 32 | 43.8 | 29.6, 64.9 | 33 | 42.2 | 28.7, 62.1 | 1.29 | 0.65, 2.59 | 0.46 | 12.5 |
| Calcium alone | 39 | 44.6 | 32.4, 61.5 | 39 | 55.8 | 40.5, 76.9 |  |  |  |  |
| Vitamin D + calcium | 35 | 43.9 | 31.3, 61.6 | 36 | 41.5 | 29.7, 58.0 | 0.76 | 0.45, 1.27 | 0.28 | -13.6 |
|  |  |  |  |  |  |  |  |  |  |  |
| *Lower 60% of crypts* |  |  |  |  |  |  |  |  |  |  |
| No vitamin D | 51 | 1086 | 974, 1211 | 51 | 1186 | 1064, 1323 |  |  |  |  |
| Vitamin D | 52 | 1137 | 1021, 1267 | 53 | 1211 | 1088, 1348 | 0.97 | 0.83, 1.15 | 0.75 | -27.1 |
| No calcium | 29 | 1241 | 1066, 1444 | 29 | 1192 | 1024, 1387 |  |  |  |  |
| Calcium | 32 | 1160 | 1005, 1339 | 33 | 1173 | 1018, 1352 | 1.05 | 0.85, 1.30 | 0.63 | 62.4 |
| Calcium alone | 39 | 1007 | 891, 1139 | 39 | 1152 | 1018, 1302 |  |  |  |  |
| Vitamin D + calcium | 35 | 1126 | 989, 1282 | 36 | 1251 | 1100, 1422 | 0.97 | 0.81, 1.17 | 0.75 | -19.8 |
|  |  |  |  |  |  |  |  |  |  |  |
| *ɸ_h_* |  |  |  |  |  |  |  |  |  |  |
| No vitamin D | 51 | 0.045 | 0.035, 0.057 | 51 | 0.044 | 0.034, 0.056 |  |  |  |  |
| Vitamin D | 52 | 0.036 | 0.028, 0.046 | 53 | 0.031 | 0.024, 0.039 | 0.86 | 0.58, 1.28 | 0.46 | -0.004 |
| No calcium | 29 | 0.042 | 0.030, 0.059 | 29 | 0.033 | 0.023, 0.046 |  |  |  |  |
| Calcium | 32 | 0.036 | 0.026, 0.050 | 33 | 0.034 | 0.025, 0.047 | 1.22 | 0.69, 2.15 | 0.49 | 0.007 |
| Calcium alone | 39 | 0.042 | 0.032, 0.055 | 39 | 0.045 | 0.034, 0.059 |  |  |  |  |
| Vitamin D + calcium | 35 | 0.037 | 0.028, 0.049 | 36 | 0.032 | 0.024, 0.042 | 0.79 | 0.51, 1.21 | 0.28 | -0.008 |
|  |  |  |  |  |  |  |  |  |  |  |
| **p21 (OD)** |  |  |  |  |  |  |  |  |  |  |
| *Whole crypts* |  |  |  |  |  |  |  |  |  |  |
| No vitamin D | 51 | 599.4 | 507.5, 708.1 | 51 | 495.1 | 419.1, 584.8 |  |  |  |  |
| Vitamin D | 53 | 603.2 | 512.2, 710.2 | 53 | 540.4 | 458.9, 636.3 | 1.08 | 0.86, 1.36 | 0.48 | 41.5 |
| No calcium | 29 | 631.1 | 519.9, 766.2 | 29 | 491.9 | 405.2, 597.1 |  |  |  |  |
| Calcium | 33 | 652.3 | 544.1, 782.0 | 33 | 585.9 | 488.7, 702.5 | 1.15 | 0.89, 1.49 | 0.27 | 72.8 |
| Calcium alone | 39 | 580.0 | 478.7, 702.6 | 39 | 464.4 | 383.3, 562.6 |  |  |  |  |
| Vitamin D + calcium | 36 | 580.1 | 475.1, 708.3 | 36 | 585.2 | 479.3, 714.6 | 1.26 | 0.96, 1.65 | 0.09 | 120.7 |
|  |  |  |  |  |  |  |  |  |  |  |
| *Upper 40% of crypts* | |  |  |  |  |  |  |  |  |  |
| No vitamin D | 51 | 318.9 | 265.8, 382.7 | 51 | 253.1 | 211.0, 303.7 |  |  |  |  |
| Vitamin D | 53 | 316.9 | 265.0, 378.9 | 53 | 281.4 | 235.3, 336.4 | 1.12 | 0.88, 1.42 | 0.35 | 30.3 |
| No calcium | 29 | 344.7 | 277.7, 427.8 | 29 | 248.6 | 200.3, 308.6 |  |  |  |  |
| Calcium | 33 | 357.5 | 292.0, 437.6 | 33 | 317.0 | 259.0, 388.1 | 1.23 | 0.93, 1.63 | 0.15 | 55.6 |
| Calcium alone | 39 | 302.5 | 245.4, 372.9 | 39 | 238.4 | 193.4, 293.9 |  |  |  |  |
| Vitamin D + calcium | 36 | 303.8 | 244.3, 377.7 | 36 | 309.3 | 248.8, 384.6 | 1.29 | 0.98, 1.70 | 0.06 | 69.6 |
|  |  |  |  |  |  |  |  |  |  |  |
| *Lower 60% of crypts* |  |  |  |  |  |  |  |  |  |  |
| No vitamin D | 51 | 37.2 | 24.8, 55.8 | 51 | 29.1 | 19.4, 43.7 |  |  |  |  |
| Vitamin D | 53 | 33.6 | 22.6, 50.0 | 53 | 28.3 | 19.0, 42.2 | 1.08 | 0.58, 1.99 | 0.81 | 2.8 |
| No calcium | 29 | 36.9 | 22.6, 60.2 | 29 | 32.8 | 20.1, 53.6 |  |  |  |  |
| Calcium | 33 | 39.3 | 24.8, 62.1 | 33 | 33.5 | 21.2, 53.0 | 0.96 | 0.48, 1.91 | 0.90 | -1.7 |
| Calcium alone | 39 | 35.7 | 22.5, 56.4 | 39 | 22.3 | 14.1, 35.2 |  |  |  |  |
| Vitamin D + calcium | 36 | 32.0 | 19.8, 51.5 | 36 | 32.1 | 19.9, 51.8 | 1.61 | 0.76, 3.39 | 0.21 | 13.5 |
|  |  |  |  |  |  |  |  |  |  |  |
| *ɸ_h_* |  |  |  |  |  |  |  |  |  |  |
| No vitamin D | 51 | 0.532 | 0.510, 0.555 | 51 | 0.511 | 0.490, 0.533 |  |  |  |  |
| Vitamin D | 53 | 0.525 | 0.504, 0.548 | 53 | 0.521 | 0.499, 0.543 | 1.03 | 0.97, 1.10 | 0.35 | 0.017 |
| No calcium | 29 | 0.546 | 0.519, 0.575 | 29 | 0.506 | 0.480, 0.532 |  |  |  |  |
| Calcium | 33 | 0.548 | 0.522, 0.575 | 33 | 0.541 | 0.515, 0.568 | 1.07 | 0.99, 1.15 | 0.08 | 0.033 |
| Calcium alone | 39 | 0.522 | 0.496, 0.548 | 39 | 0.513 | 0.489, 0.540 |  |  |  |  |
| Vitamin D + calcium | 36 | 0.524 | 0.497, 0.552 | 36 | 0.529 | 0.502, 0.557 | 1.03 | 0.95, 1.11 | 0.53 | 0.014 |
|  |  |  |  |  |  |  |  |  |  |  |
| **p21/mib-1 (OD)** |  |  |  |  |  |  |  |  |  |  |
| *Whole crypts* |  |  |  |  |  |  |  |  |  |  |
| No vitamin D | 51 | 0.52 | 0.45, 0.60 | 51 | 0.39 | 0.34, 0.45 |  |  |  |  |
| Vitamin D | 53 | 0.50 | 0.44, 0.58 | 53 | 0.42 | 0.37, 0.49 | 1.13 | 0.90, 1.42 | 0.30 | 0.05 |
| No calcium | 29 | 0.50 | 0.42, 0.59 | 29 | 0.40 | 0.34, 0.48 |  |  |  |  |
| Calcium | 33 | 0.54 | 0.47, 0.61 | 33 | 0.42 | 0.37, 0.47 | 0.95 | 0.74, 1.23 | 0.72 | -0.02 |
| Calcium alone | 39 | 0.54 | 0.46, 0.64 | 39 | 0.38 | 0.32, 0.45 |  |  |  |  |
| Vitamin D + calcium | 36 | 0.49 | 0.41, 0.58 | 36 | 0.45 | 0.37, 0.53 | 1.33 | 1.00, 1.75 | 0.05 | 0.12 |
|  |  |  |  |  |  |  |  |  |  |  |
| *Upper 40% of crypts* |  |  |  |  |  |  |  |  |  |  |
| No vitamin D | 51 | 6.21 | 4.51, 8.56 | 51 | 4.57 | 3.32, 6.31 |  |  |  |  |
| Vitamin D | 53 | 7.25 | 5.29, 9.93 | 53 | 7.24 | 5.29, 9.92 | 1.36 | 0.82, 2.26 | 0.24 | 1.63 |
| No calcium | 29 | 6.54 | 4.27, 10.02 | 29 | 6.34 | 4.14, 9.72 |  |  |  |  |
| Calcium | 33 | 6.76 | 4.97, 9.19 | 33 | 5.33 | 3.92, 7.24 | 0.81 | 0.44, 1.51 | 0.51 | -1.23 |
| Calcium alone | 39 | 6.78 | 4.72, 9.72 | 39 | 4.27 | 2.98, 6.13 |  |  |  |  |
| Vitamin D + calcium | 36 | 6.81 | 4.68, 9.92 | 36 | 7.45 | 5.12, 10.85 | 1.73 | 0.98, 3.06 | 0.06 | 3.15 |
|  |  |  |  |  |  |  |  |  |  |  |
| *Lower 60% of crypts* |  |  |  |  |  |  |  |  |  |  |
| No vitamin D | 51 | 0.034 | 0.023, 0.050 | 51 | 0.025 | 0.017, 0.036 |  |  |  |  |
| Vitamin D | 53 | 0.029 | 0.020, 0.043 | 53 | 0.023 | 0.016, 0.034 | 1.11 | 0.62, 1.98 | 0.72 | 0.003 |
| No calcium | 29 | 0.311 | 0.019, 0.051 | 29 | 0.029 | 0.018, 0.047 |  |  |  |  |
| Calcium | 33 | 0.034 | 0.024, 0.048 | 33 | 0.022 | 0.016, 0.032 | 0.72 | 0.37, 1.37 | 0.31 | 0.270 |
| Calcium alone | 39 | 0.035 | 0.023, 0.055 | 39 | 0.019 | 0.013, 0.030 |  |  |  |  |
| Vitamin D + calcium | 36 | 0.028 | 0.018, 0.044 | 36 | 0.026 | 0.016, 0.040 | 1.67 | 0.81, 3.43 | 0.16 | 0.014 |

Abbreviations: Abs, absolute treatment effect; OD, optical density; 95% CI, 95% confidence interval

^a^ Presented as geometric means and 95% confidence intervals.

^b^ Relative treatment effect from SAS Institute's Mixed Procedure defined as [(active treatment group follow-up mean) / (active treatment group baseline mean)] / [(control group follow-up mean) / (control group baseline mean)].

^c^ *P* value for difference between each active treatment group and control group from repeated-measures MIXED model

^d^ Absolute treatment effect calculated as [(active treatment group follow-up mean) - (active treatment group baseline mean)] - [(control group follow-up mean) - (control group baseline mean)]
