# Supplementary material for: Effects of supplemental vitamin D and calcium on markers of proliferation, differentiation, and apoptosis in the normal colorectal mucosa of colorectal adenoma patients
Source: PLoS One. 2018 Dec 17;13(12):e0208762. doi: 10.1371/journal.pone.0208762 (PMC6296527; doi:10.1371/journal.pone.0208762)
Supplement: S2 Table — (DOCX) [file pone.0208762.s002.docx]

**Supplemental Table 2. Changes in biomarkers of apoptosis in colorectal crypts of the adjunct biomarker study participants (n = 104)^a^.**

|  | **Baseline** | | | **1-Year follow-up** | | | **Treatment effect** | | | |
| --- | --- | --- | --- | --- | --- | --- | --- | --- | --- | --- |
| **Treatment group** | **n** | **Mean** | **95% CI** | **n** | **Mean** | **95% CI** | **Relative**^b^ | **95% CI** | ***P****^c^* | **Abs**^d^ |
| **bax (OD)** |  |  |  |  |  |  |  |  |  |  |
| *Whole crypts* |  |  |  |  |  |  |  |  |  |  |
| No vitamin D | 51 | 275.6 | 218.4, 347.8 | 51 | 381.9 | 302.6, 482.0 |  |  |  |  |
| Vitamin D | 53 | 296.2 | 235.8, 372.3 | 53 | 397.3 | 316.2, 499.2 | 0.97 | 0.68, 1.38 | 0.86 | -5.2 |
| No calcium | 29 | 289.3 | 208.8, 400.9 | 29 | 409.9 | 295.8, 567.9 |  |  |  |  |
| Calcium | 33 | 295.1 | 217.5, 400.4 | 33 | 345.6 | 254.7, 468.9 | 0.83 | 0.52, 1.32 | 0.42 | -70.1 |
| Calcium alone | 39 | 292.6 | 222.1, 385.5 | 39 | 350.9 | 266.4, 462.3 |  |  |  |  |
| Vitamin D + calcium | 36 | 270.7 | 203.2, 360.6 | 36 | 410.7 | 308.2, 547.2 | 1.27 | 0.83, 1.92 | 0.26 | 81.8 |
|  |  |  |  |  |  |  |  |  |  |  |
| *Upper 40% of crypts* |  |  |  |  |  |  |  |  |  |  |
| No vitamin D | 51 | 68.8 | 51.5, 91.8 | 51 | 111.4 | 83.4, 148.7 |  |  |  |  |
| Vitamin D | 53 | 81.9 | 61.7, 108.8 | 53 | 114.1 | 85.9, 151.6 | 0.86 | 0.57, 1.29 | 0.47 | -10.4 |
| No calcium | 29 | 73.8 | 48.5, 112.3 | 29 | 120.4 | 79.1, 183.2 |  |  |  |  |
| Calcium | 33 | 74.2 | 50.1, 110.0 | 33 | 101.8 | 69.7, 150.8 | 0.84 | 0.49, 1.43 | 0.51 | -19.0 |
| Calcium alone | 39 | 77.2 | 55.0, 108.5 | 39 | 103.8 | 73.8, 145.8 |  |  |  |  |
| Vitamin D + calcium | 36 | 73.5 | 51.6, 104.8 | 36 | 116.1 | 81.5, 165.5 | 1.18 | 0.71, 1.95 | 0.53 | 16.0 |
|  |  |  |  |  |  |  |  |  |  |  |
| *Lower 60% of crypts* |  |  |  |  |  |  |  |  |  |  |
| No vitamin D | 51 | 180.2 | 142.6, 227.5 | 51 | 242.9 | 192.3, 306.8 |  |  |  |  |
| Vitamin D | 53 | 187.5 | 149.1, 235.8 | 53 | 247.9 | 197.1, 311.7 | 0.98 | 0.68, 1.42 | 0.92 | -2.3 |
| No calcium | 29 | 189.7 | 137.8, 261.2 | 29 | 257.1 | 186.8, 353.9 |  |  |  |  |
| Calcium | 33 | 192.3 | 142.6, 259.4 | 33 | 209.2 | 155.1, 282.1 | 0.80 | 0.49, 1.31 | 0.37 | -50.5 |
| Calcium alone | 39 | 187.0 | 141.8, 246.6 | 39 | 221.6 | 168.1, 292.2 |  |  |  |  |
| Vitamin D + calcium | 36 | 171.7 | 128.7, 228.9 | 36 | 257.5 | 193.1, 343.5 | 1.27 | 0.82, 1.95 | 0.28 | 51.2 |
|  |  |  |  |  |  |  |  |  |  |  |
| *ɸ_h_* |  |  |  |  |  |  |  |  |  |  |
| No vitamin D | 51 | 0.249 | 0.221, 0.282 | 51 | 0.292 | 0.258, 0.330 |  |  |  |  |
| Vitamin D | 53 | 0.277 | 0.245, 0.312 | 53 | 0.287 | 0.255, 0.324 | 0.89 | 0.73, 1.09 | 0.25 | -0.033 |
| No calcium | 29 | 0.255 | 0.214, 0.304 | 29 | 0.294 | 0.247, 0.350 |  |  |  |  |
| Calcium | 33 | 0.252 | 0.214, 0.296 | 33 | 0.294 | 0.250, 0.347 | 1.02 | 0.77, 1.35 | 0.91 | 0.003 |
| Calcium alone | 39 | 0.264 | 0.230, 0.303 | 39 | 0.296 | 0.257, 0.340 |  |  |  |  |
| Vitamin D + calcium | 36 | 0.272 | 0.235, 0.314 | 36 | 0.283 | 0.245, 0.327 | 0.93 | 0.72, 1.19 | 0.56 | -0.021 |
|  |  |  |  |  |  |  |  |  |  |  |
| **bcl-2 (OD)** |  |  |  |  |  |  |  |  |  |  |
| *Whole crypts* |  |  |  |  |  |  |  |  |  |  |
| No vitamin D | 51 | 669.8 | 573.9, 781.7 | 51 | 706.7 | 605.5, 824.8 |  |  |  |  |
| Vitamin D | 52 | 694.4 | 595.9, 809.3 | 52 | 752.4 | 645.7, 876.7 | 1.03 | 0.81, 1.30 | 0.82 | 21.1 |
| No calcium | 29 | 631.8 | 516.7, 772.6 | 29 | 693.6 | 567.2, 848.2 |  |  |  |  |
| Calcium | 32 | 724.8 | 598.7, 877.5 | 32 | 701.7 | 579.6, 849.5 | 0.88 | 0.68, 1.15 | 0.34 | -84.9 |
| Calcium alone | 39 | 671.8 | 560.7, 804.9 | 39 | 688.1 | 574.3, 824.5 |  |  |  |  |
| Vitamin D + calcium | 35 | 717.5 | 592.8, 868.4 | 35 | 787.5 | 650.6, 953.1 | 1.07 | 0.79, 1.44 | 0.65 | 53.7 |
|  |  |  |  |  |  |  |  |  |  |  |
| *Upper 40% of crypts* |  |  |  |  |  |  |  |  |  |  |
| No vitamin D | 51 | 35.0 | 26.9, 45.6 | 51 | 38.2 | 29.3, 49.7 |  |  |  |  |
| Vitamin D | 52 | 39.7 | 30.5, 51.5 | 52 | 38.7 | 29.8, 50.3 | 0.90 | 0.59, 1.37 | 0.61 | -4.2 |
| No calcium | 29 | 40.3 | 28.6, 56.8 | 29 | 37.2 | 26.4, 52.4 |  |  |  |  |
| Calcium | 32 | 31.0 | 22.4, 42.9 | 32 | 30.3 | 21.9, 41.9 | 1.06 | 0.66, 1.70 | 0.81 | 2.4 |
| Calcium alone | 39 | 34.1 | 24.8, 46.8 | 39 | 36.9 | 26.9, 50.7 |  |  |  |  |
| Vitamin D + calcium | 35 | 37.9 | 27.1, 53.0 | 35 | 40.6 | 29.0, 56.8 | 0.99 | 0.58, 1.68 | 0.97 | -0.1 |
|  |  |  |  |  |  |  |  |  |  |  |
| *Lower 60% of crypts* |  |  |  |  |  |  |  |  |  |  |
| No vitamin D | 51 | 614.1 | 525.7, 717.5 | 51 | 654.3 | 560.1, 764.4 |  |  |  |  |
| Vitamin D | 52 | 636.2 | 545.4, 742.1 | 52 | 696.2 | 596.9, 812.2 | 1.03 | 0.82, 1.29 | 0.82 | 19.8 |
| No calcium | 29 | 566.6 | 460.5, 696.9 | 29 | 637.3 | 518.1, 784.1 |  |  |  |  |
| Calcium | 32 | 678.7 | 557.4, 826.4 | 32 | 660.2 | 542.1, 803.8 | 0.86 | 0.66, 1.13 | 0.28 | -89.2 |
| Calcium alone | 39 | 625.1 | 522.5, 747.7 | 39 | 638.1 | 533.4, 763.3 |  |  |  |  |
| Vitamin D + calcium | 35 | 657.1 | 543.9, 793.9 | 35 | 730.4 | 604.6, 882.5 | 1.09 | 0.82, 1.45 | 0.55 | 60.3 |
|  |  |  |  |  |  |  |  |  |  |  |
| *ɸ_h_* |  |  |  |  |  |  |  |  |  |  |
| No vitamin D | 51 | 0.052 | 0.042, 0.065 | 51 | 0.054 | 0.044, 0.067 |  |  |  |  |
| Vitamin D | 52 | 0.057 | 0.046, 0.070 | 52 | 0.051 | 0.042, 0.063 | 0.87 | 0.66, 1.15 | 0.33 | -0.008 |
| No calcium | 29 | 0.064 | 0.047, 0.087 | 29 | 0.054 | 0.039, 0.073 |  |  |  |  |
| Calcium | 32 | 0.043 | 0.032, 0.057 | 32 | 0.043 | 0.032, 0.058 | 1.20 | 0.85, 1.70 | 0.29 | 0.010 |
| Calcium alone | 39 | 0.051 | 0.040, 0.065 | 39 | 0.054 | 0.042, 0.069 |  |  |  |  |
| Vitamin D + calcium | 35 | 0.053 | 0.041, 0.069 | 35 | 0.052 | 0.040, 0.067 | 0.92 | 0.66, 1.28 | 0.63 | -0.004 |
|  |  |  |  |  |  |  |  |  |  |  |
| **bax/bcl-2 (OD)** |  |  |  |  |  |  |  |  |  |  |
| *Whole crypts* |  |  |  |  |  |  |  |  |  |  |
| No vitamin D | 51 | 0.41 | 0.33, 0.52 | 51 | 0.54 | 0.43, 0.68 |  |  |  |  |
| Vitamin D | 52 | 0.44 | 0.35, 0.55 | 52 | 0.53 | 0.42, 0.66 | 0.92 | 0.66, 1.29 | 0.64 | -0.04 |
| No calcium | 29 | 0.45 | 0.33, 0.62 | 29 | 0.58 | 0.43, 0.80 |  |  |  |  |
| Calcium | 32 | 0.46 | 0.36, 0.57 | 32 | 0.53 | 0.42, 0.66 | 0.90 | 0.60, 1.34 | 0.59 | -0.06 |
| Calcium alone | 39 | 0.44 | 0.33, 0.57 | 39 | 0.51 | 0.39, 0.66 |  |  |  |  |
| Vitamin D + calcium | 35 | 0.39 | 0.29, 0.51 | 35 | 0.52 | 0.39, 0.69 | 1.15 | 0.79, 1.68 | 0.47 | 0.06 |
|  |  |  |  |  |  |  |  |  |  |  |
| *Upper 40% of crypts* |  |  |  |  |  |  |  |  |  |  |
| No vitamin D | 51 | 1.96 | 1.41, 2.74 | 51 | 2.92 | 2.09, 4.08 |  |  |  |  |
| Vitamin D | 52 | 2.13 | 1.53, 2.96 | 52 | 2.89 | 2.08, 4.03 | 0.92 | 0.60, 1.40 | 0.68 | -0.20 |
| No calcium | 29 | 1.81 | 1.16, 2.83 | 29 | 3.20 | 2.05, 5.00 |  |  |  |  |
| Calcium | 32 | 2.48 | 1.79, 3.43 | 32 | 3.09 | 2.23, 4.27 | 0.70 | 0.43, 1.15 | 0.16 | -0.78 |
| Calcium alone | 39 | 2.27 | 1.55, 3.32 | 39 | 2.81 | 1.92, 4.12 |  |  |  |  |
| Vitamin D + calcium | 35 | 2.02 | 1.35, 3.02 | 35 | 2.79 | 1.86, 4.17 | 1.11 | 0.66, 1.89 | 0.69 | 0.23 |
| *Lower 60% of crypts* |  |  |  |  |  |  |  |  |  |  |
| No vitamin D | 51 | 0.29 | 0.23, 0.37 | 51 | 0.37 | 0.30, 0.47 |  |  |  |  |
| Vitamin D | 52 | 0.30 | 0.24, 0.38 | 52 | 0.36 | 0.29, 0.46 | 0.96 | 0.67, 1.38 | 0.82 | -0.02 |
| No calcium | 29 | 0.33 | 0.24, 0.45 | 29 | 0.40 | 0.29, 0.54 |  |  |  |  |
| Calcium | 32 | 0.32 | 0.25, 0.40 | 32 | 0.36 | 0.28, 0.45 | 0.94 | 0.61, 1.44 | 0.76 | -0.03 |
| Calcium alone | 39 | 0.30 | 0.23, 0.39 | 39 | 0.35 | 0.27, 0.45 |  |  |  |  |
| Vitamin D + calcium | 35 | 0.27 | 0.20, 0.35 | 35 | 0.36 | 0.28, 0.48 | 1.18 | 0.78, 1.77 | 0.43 | 0.04 |
|  |  |  |  |  |  |  |  |  |  |  |
| **bax/mib-1 (OD)** |  |  |  |  |  |  |  |  |  |  |
| *Whole crypts* |  |  |  |  |  |  |  |  |  |  |
| No vitamin D | 51 | 0.24 | 0.19, 0.30 | 51 | 0.30 | 0.24, 0.38 |  |  |  |  |
| Vitamin D | 53 | 0.25 | 0.19, 0.31 | 53 | 0.31 | 0.25, 0.40 | 1.01 | 0.68, 1.48 | 0.98 | 0.00 |
| No calcium | 29 | 0.22 | 0.16, 0.31 | 29 | 0.33 | 0.24, 0.46 |  |  |  |  |
| Calcium | 33 | 0.26 | 0.21, 0.33 | 33 | 0.29 | 0.23, 0.36 | 0.75 | 0.47, 1.18 | 0.21 | -0.08 |
| Calcium alone | 39 | 0.27 | 0.21, 0.36 | 39 | 0.28 | 0.22, 0.37 |  |  |  |  |
| Vitamin D + calcium | 36 | 0.23 | 0.17, 0.30 | 36 | 0.31 | 0.23, 0.42 | 1.33 | 0.85, 2.08 | 0.21 | 0.07 |
|  |  |  |  |  |  |  |  |  |  |  |
| *Upper 40% of crypts* |  |  |  |  |  |  |  |  |  |  |
| No vitamin D | 51 | 1.34 | 0.89, 2.01 | 51 | 2.01 | 1.34, 3.02 |  |  |  |  |
| Vitamin D | 53 | 1.87 | 1.26, 2.79 | 53 | 2.94 | 1.98, 4.37 | 1.04 | 0.54, 2.01 | 0.90 | 0.40 |
| No calcium | 29 | 1.36 | 0.79, 2.34 | 29 | 2.97 | 1.72, 5.13 |  |  |  |  |
| Calcium | 33 | 1.60 | 1.08, 2.37 | 33 | 2.07 | 1.40, 3.07 | 0.59 | 0.26, 1.33 | 0.20 | -1.14 |
| Calcium alone | 39 | 1.73 | 1.10, 2.72 | 39 | 1.86 | 1.18, 2.92 |  |  |  |  |
| Vitamin D + calcium | 36 | 1.65 | 1.03, 2.64 | 36 | 2.80 | 1.75, 4.48 | 1.58 | 0.77, 3.24 | 0.21 | 1.02 |
|  |  |  |  |  |  |  |  |  |  |  |
| *Lower 60% of crypts* |  |  |  |  |  |  |  |  |  |  |
| No vitamin D | 51 | 0.17 | 0.13, 0.21 | 51 | 0.20 | 0.16, 0.36 |  |  |  |  |
| Vitamin D | 53 | 0.16 | 0.13, 0.21 | 53 | 0.20 | 0.16, 0.26 | 1.01 | 0.68, 1.50 | 0.96 | 0.01 |
| No calcium | 29 | 0.16 | 0.11, 0.21 | 29 | 0.22 | 0.16, 0.30 |  |  |  |  |
| Calcium | 33 | 0.18 | 0.14, 0.22 | 33 | 0.19 | 0.15, 0.24 | 0.75 | 0.47, 1.20 | 0.22 | -0.05 |
| Calcium alone | 39 | 0.19 | 0.14, 0.24 | 39 | 0.19 | 0.15, 0.25 |  |  |  |  |
| Vitamin D + calcium | 36 | 0.15 | 0.11, 0.20 | 36 | 0.21 | 0.16, 0.27 | 1.31 | 0.83, 2.08 | 0.24 | 0.06 |

Abbreviations: Abs, absolute treatment effect; OD, optical density; 95% CI, 95% confidence interval

^a^ Presented as geometric means and 95% confidence intervals.

^b^ Relative treatment effect from SAS Institute's Mixed Procedure defined as [(active treatment group follow-up mean) / (active treatment group baseline mean)] / [(control group follow-up mean) / (control group baseline mean)].

^c^ *P* value for difference between each active treatment group and control group from repeated-measures MIXED model

^d^ Absolute treatment effect calculated as [(active treatment group follow-up mean) - (active treatment group baseline mean)] - [(control group follow-up mean) - (control group baseline mean)]
